# Safety and efficacy of superior calyceal access versus inferior calyceal access for pelvic and/or lower calyceal renal calculi- a prospective observational comparative study

**DOI:** 10.1007/s00345-020-03409-3

**Published:** 2020-08-31

**Authors:** M. Amaresh, P. Hegde, A. Chawla, J. J. M. C. H. de la Rosette, M. P. Laguna, A. Kriplani

**Affiliations:** 1 Department of Urology, Kasturba Medical College, Manipal, Karnataka India; 2 Department of Urology, Istanbul Medipol University Hospital, Istanbul, Turkey

**Keywords:** PCNL, Renal, Calculi, Percutaneous nephrolithotomy, Superior calyx, Inferior calyx, Access

## Abstract

**Objective:**

To compare efficacy and safety between superior calyceal access and inferior calyceal access for pelvic and/or lower calyceal renal stones.

**Methods:**

Consecutive patients presenting with Pelvic and/or inferior calyceal renal calculi were allocated to the superior calyceal access (group 1) or inferior calyceal access (group 2) treatment arm. Allocation of treatment access was based on the surgeon’s preference. Variables studied included stone free rate, operating time, intraoperative and postoperative complications. Statistical analysis was executed using SPSS, Version 16.0. The statistical significance was evaluated at 5% level of significance (*p *value < 0.05).

**Results:**

Between July 2018 and February 2019, 63 patients were included in each group. The percutaneous inserted guidewire entered the ureter in 92% in group1 and 74.6% in group 2 (*p* = 0.034). Stone fragments migrated to the middle calyx in 3.2% in group1 and 9.5% in group 2 (*p* = 0.033). A second puncture was required in one patient in group 1 and in 5 patients in group 2 (*p* = 0.04). The operative duration (minutes) was 13.46 ± 1.09 in the group 1 while 16.58 ± 1.44 in the group 2 (*p* = 0.002). Thoracic complications (hydropneumothorax) occurred to 2 patients in superior calyceal access group managed with intercostal tube drainage (*p* = 0.243).Post operatively blood transfusion was required in two patients in group 2 (*p* = 0.169). Angioembolization was done in one patient among the inferior calyceal access approach (*p* = 0.683). Complete stone clearance assessed at 3 months was 96.8% in group 1 and 85.7% in group 2 (*p* = 0.046).

**Conclusions:**

Superior calyceal access is a safe and most efficacious in terms of achieving complete stone clearance rate with reduced operative time, minimal blood loss, less need for a second puncture and auxiliary procedures at minimal complications.

**Study registration:**

Clinical trials registry – INDIA; CTRI/2018/07/014,687.

## Introduction

With the advent of miniaturized access tracts and thereby minimal postoperative complications, PCNL dominates the treatment modalities for larger sized and/or complex renal stone management [1]. One of the major advantages of PCNL over other techniques is the higher stone clearance [2]. A key requisite to a successful procedure is establishing an optimal direct and safe access to the intrarenal collecting system facilitating ease of intrarenal navigation resulting in complete stone clearance.

Traditionally, the posterior inferior calyceal access is considered as the safest percutaneous access to the intrarenal collecting system and is widely practiced by the majority for stones in the renal pelvis, lower calyx and staghorn stones [2–4]. However, this may not be an ideal access for urinary stone removal in all circumstances. Superior calyceal access is often underutilized due to the fear of performing an intercostal puncture, risk of possible bleeding and thoracic complications[ 5].

This study was conducted to prospectively evaluate the outcomes of superior calyceal access (Group 1) and inferior calyceal access (Group 2) in PCNL performed for pelvic and/or inferior calyceal stones. The primary objective was to study the safety and stone clearance in both groups, while the secondary objective aimed to study the technical ease, blood loss, operative time, requirement of additional tracts and auxiliary procedures.

## Materials and methods

A prospective, single center, observational study, in consecutive patients diagnosed with renal calculi and scheduled for PCNL. Inclusion criteria are patients with any pelvic calculus and/or inferior calyceal calculi. Patients with staghorn calculi, isolated middle calyceal calculi or stones in calyceal diverticula were excluded. The study was approved by our Medical Ethical committee, and all patients included signed informed consent.

Each group had an equal distribution, recruited as per the operating surgeon’s preference. After a thorough history taking and clinical examination, all patients underwent renal ultrasound, X-ray KUB, NCCT KUB and blood investigations (complete blood count, renal function test, serum electrolytes, coagulation profile and serology), urine microscopy and urine culture.

Patients were treated under general anesthesia. Patients received intravenous cefoperazone during induction and were placed in the lithotomy position. Cystoscopy was performed and a 5Fr/6Fr ureteral catheter was positioned in the pelvis and secured to a Foley transurethral catheter. Next the patient was placed in the prone position. Case selection for Superior or Inferior calyceal access was decided by the operating surgeon based on findings of the intrarenal anatomy on retrograde pyelogram. After retrograde contrast injection, the selected calyx was punctured under fluoroscopic guidance. An 18 G puncture needle (Blueneem medical devices pvt ltd) was used to access the collecting system by the ‘bull’s eye’ technique followed by placement of a super stiff guidewire (Boston Scientific, USA).

Access tracts were dilated with sequential coaxial metal dilators in standard PCNL (Alken) and single step dilatation in mini PCNL (Karl Storz, Tuttlingen, Germany). After adequate dilatation an Amplatz working sheath is secured and the dilatation assembly is removed. In standard PCNL stone fragmentation is done by laser or lithotripter (Swiss lithoclast master, Electro medical systems, SA) and retrieved with triradiate forceps, whereas in mini PCNL stone was pulverized with laser (Auriga XL, Boston Scientific, USA). A 6 Fr double-J stent (Blueneem medical devices pvt ltd) was placed antegrade once fluoroscopic and endoscopic stone clearance is ensured.

Data were recorded on patient demographics, stone size, stone density in HU, puncture of collecting system (supra or infracostal), access(superior/inferior), operative time, fluoroscopy time, intraoperative findings, change in Hb and PCV (preoperatively versus 12 h post operatively), length of hospital stay, analgesic requirements, pain score, and complications. The patient was monitored for postoperative complications and graded according to the modified Clavien grading system. Patients were followed up after 1 month of surgery with X-ray KUB and renal ultrasound prior to the double-J stent removal. At the third month, sixth month, and 1 year, patients were followed with X-ray KUB and renal ultrasound.

### Statistical analysis

The sample size was calculated using PASS software, with a Power of 80%, Significance of 0.05 and level of confidence of 95%. The statistical analysis was executed using SPSS, Version 16.0. Descriptive statistics such as frequencies and percentages, mean and standard deviation were calculated. The means of Continuous variables were tested against two groups using unpaired *t* test (Independent *t* test). The Categorical variables were cross tabulated against group 1 and group 2 using Chi-square test for independence. Fisher’s exact test was applied, where 20% of expected values were less than 5. The statistical significance was evaluated at 5% level of significance (*p* value < 0.05).

## Results

From July 2018 to February 2019, 126 patients undergoing PCNL were enrolled with equal distribution (63 patients) in the superior calyceal access (group I) and inferior calyceal access (group II) groups. The preoperative variables studied are shown in Table 1.

**Table 1:** Preoperative variables

	Group I	Group II
*Patient characteristics*		
Number of patients	63	63
Mean age (years)	Range 45.81 ± 3.72	Range 46.6 ± 3.41
Sex	M-38 F-25	M-49 F-14
BMI (kg/m^2^)	< 18.5: 13 18.5–24.9: 30 > 25: 20	< 18.5: 15 18.5–24.9: 27 > 25: 21
*Stone characteristics*		
Mean (SD) Stone burden (cm^2^)	Range 2.54 (0.3)	Range 1.97 (0.36)
Stone position	Pelvis: 32 Inferior calyx: 18 Both: 13	Pelvis: 36 Inferior calyx: 20 Both: 7
Mean (SD) stone density (HU)	725.8 (0.78)	738.9 (0.77)

Demographic and preoperative characteristics of the patients in the two groups were comparable. The mean BMI in group I was 22.71 kg/m^2^ and group II was 22.74 kg/m^2^ with comparable BMI and body habitus and, therefore, skin to stone distance between the two groups. Stone characteristics in relation to calculus size, position and density were similar in the two groups, with the majority presenting as pelvic calculi followed by inferior calyceal calculi.

Instrumentation used included access sheath sizes ranging from 12Fr up to 32 Fr and. Nephroscope sizes ranged from 7.5Fr to 26Fr (Table 2). Intraoperative variables recorded are tabulated in Table 3. Easy navigation of the guidewire into the collecting system and ureter after primary puncture was possible in 92.06% patients in group I, while 25.39% (*n* = 16) patients in group II faced difficulty in negotiating the guidewire due to stones obstructing the lower calyx. Kinking, bending, slippage of the guidewire was observed in 14.28% (*n* = 9) subjects in group II, while no such instances were observed in group 1. A repeat puncture was required in 6.34% (*n* = 4) subjects in group II due to guidewire dislodgement from the collecting system. During fragmentation Stone migration from pelvis to distant calyces was seen in 9.52% patients was significantly more in group II (*p* = 0.033). Need for a second puncture to achieve complete stone clearance was significantly higher in group II. One patient in each group was managed with a secondary needle puncture and saline irrigation pushing the stone back to the pelvis and removing it from the primary tract. Operative time, defined as the time taken from the puncture of kidney to removal of Amplatz sheath after completion of stone fragmentation, was significantly lower in group I (*p* = 0.002).Fluoroscopy time, defined as the total duration of fluoroscopy exposure during the puncture, dilation, lithotripsy till complete fragmentation, was comparable in the two groups. Torque, defined as the rotational force exerted by the nephroscope on the renal parenchyma during navigation from the primary tract axis to visualize and fragment the stone, causing calyceal or infundibular injury leading to increased bleeding, was significantly lower in group I.

**Table 2:** Instrumentation used in the two groups

	Group I			Group II		
	Size (Fr)	Frequency	%	Frequency	%	*P*-value
Amplatz size	12	6	9.5	3	4.8	0.106
	24	1	1.6	1	1.6	
	26	5	7.9	8	12.7	
	28	15	23.8	24	38.1	
	30	13	17.5	16	23.8	
	32	23	36.5	11	17.5	
Nephroscope size	7.5	6	9.5	3	4.8	0.230
	20.8	18	28.6	27	42.9	
	26	39	61.9	33	52.4

**Table 3:** Intraoperative characteristics

	Group I	Group II	*P*-value
Supracostal puncture	34	3	
Infracostal puncture	29	60	
PCNL guidewire entering the ureter	58	47	0.034
Stone migration	2	6	0.033
Second puncture for migrated stone	1	5	0.04
Torque	Present: 4	Present: 11	
	Absent: 59	Absent: 52	
Mean (SD) operative time (Min.sec)	13.96 (1.09)	16.58 (1.44)	0.002
Mean (SD) fluoroscopy (Min.sec)	4.30 (0.30)	4.45 (0.58)	0.418
Calyceal injury	Present- 2 Absent- 61	Present- 12 Absent- 51	0.002

Post-operative pain was assessed on Visual Analogue Scale (Static and Dynamic) ranging from 0 to 10. Intravenous analgesic Tramadol was given on-demand depending on the pain score. Both superior calyceal and inferior calyceal access groups had comparable pain scores at 1 h, 6 h and 24 h (Table 4). Postoperatively assessed parameters are tabulated in Table 5. Drop in postoperative hemoglobin (*P* = 0.039) and hematocrit (*P *< 0.001) was significantly more in Group II. One patient in group II had persistent hematuria and hypotension in the postoperative period. He was diagnosed to have developed a pseudo aneurysm of the inferior polar artery and underwent angioembolization. Postoperative complications classified by the modified Clavien Grading System [14] included transient low-grade fever which only required observation (Table 6). Postoperative UTI was managed by culture specific antibiotics. 4.7% patients in group II had significant blood loss and required a single unit of blood transfusion. Both these patients underwent cystoscopy and sheath wash for clot retention postoperatively. In group I, 3.1% patients who had a supracostal puncture developed pleural injury leading to hydro-pneumothorax. These were diagnosed on clinical suspicion and chest radiography postoperatively and were managed with an intercostal chest tube drainage. Intercostal tube drain was removed within 12 h in one patient and within 24 h in the other. None of the patients had to stay longer because of morbidity of intercostal drainage.

**Table 4:** Postoperative pain score (VAS)

	(Group I)		(Group II)		*P* value
	Mean (SD)	95% CI	Mean (SD)	95% CI	
Pain at 1 h	5.34 (0.80)	5.1–45.54	5.50 (0.91)	5.28–5.73	0.303
Pain at 6 h	3.69 (0.77)	3.50–3.88	3.82 (0.97)	3.58–4.06	0.420
Pain at 24 h	2.54 (0.56)	2.41–2.68	2.79 (0.78)	2.60–3.0	0.825

**Table 5:** Postoperative variables

	Group I	Group II	*P*-value
Mean (SD) Hb difference (gm/dl)	1.31 (0.06)	1.46 (0.03)	0.039
Mean (SD) PCV difference	4.63 (0.23)	3.52 (0.28)	< 0.001
Mean (SD) hospital stay (days)	2.36 (0.66)	2.55(0.72)	0.70
Residual calculi at 1 month (USG/X-Ray)	Present 2 Absent 61	Present 9 Absent 54	0.002
*Need for auxiliary procedures*			
Yes	*N* = 1	*N* = 2	
No	*N* = 62	*N* = 61

**Table 6:** Complications (modified clavien grading system)

Class		SCA (Group I)	ICA (Group II)	*P* value
I	Transient postop Fever	1 (1.5%)	2 (3.1%)	0.086
II	Bleeding requiring transfusion	0	2 (3.1%)	0.169
	UTI managed with antibiotics	0	1 (1.5%)	0.063
III	Pneumothorax with chest tube	2 (3.1%)	0	0.243
	Clot retention	0	2 (3.1%)	0.058

Duration of hospital stay defined as the time from the start of procedure to the time postoperatively when the patient is deemed to be fit (decreased pain, ambulatory, tolerating oral feeds, can take care of oneself independently) for discharge by the operating surgeon. Both groups had a comparable duration of hospital stay.

Clinically significant residual fragments (CSRFs) are defined as any fragment sized above 4 mm on ultrasound / X-ray KUB. Complete stone clearance is defined as absence of clinically significant residual fragments (> 4 mm) at 1 month and 3 months postoperatively on X-ray KUB and renal USG. There was a significant difference observed among the two groups, with complete stone clearance significantly higher in group I as compared to group II (96.82% versus 85.71%) (*P* = 0.002). Effect of Tract size on stone free rates are compared and tabulated in Table 7. One patient in group I and 7 patients in group II had ≤ 7 mm residual calculi in the kidney. These patients were given the option for ESWL/RIRS during stent removal. One patient in the group I and two patients in group II had radiopaque shadows in the ureter along the double-J stent at 1 month on X-ray KUB. All 3 patients in both the groups required ureteroscopy as auxiliary procedure at the time of stent removal. There was no significant statistical difference noted between the two groups. Stone free rates comparing isolated pelvic stone in both groups were 98.5% in group I and 95.7% in group II (*p* = 0.032) and in isolated lower pole calculus was 92.5% and 82.7% in group I and II (*p* = 0.002), respectively (Table 8).

**Table 7:** Tract size and stone free rate in both the groups

Tract size (Fr)	Group I (SFR %)	Group II (SFR %)
12	95	92
24	96	85
26	95	84
28	97	85
30	99	86
32	99	82
	96.8	85.7

**Table 8:** Stone location and stone free rate in both the groups

Stone location	Group 1 (SCA) *N* = 63	Stone free rate (%)	Group 2 (ICA) *N* = 63	Stone free rate (%)	*p* value
Pelvis	32 (50.8%)	98.5	36 (57.1%)	92.5	0.032
Inferior calyx	18 (28.6%)	95.7	20 (31.7%)	82.7	0.002
Pelvis + Inferior calyx	13 (20.6%)	94.8	7 (11.1%)	81.9	0.013

## Discussion

We are witnessing a renaissance in percutaneous stone surgery fueled by miniaturization of instruments, position during surgery and choice of calyceal access. This observational study elegantly confirms that the superior pole access has significant benefits over inferior pole access in patients with renal pelvis stones and/or lower pole stones. In line with the literature, the stone free rate is higher in superior pole access at a lower morbidity [3–7]*.* However, this is one of the first studies that have evaluated this in a prospective study.

Obviously, the choice of access is crucial and has resulted in a strong debate about the choice of access. Traditionally the choice is limited between the superior and inferior calyx access. That is why we have limited our choice of access accordingly. With the introduction of mini PCNL, however, also the middle pole access has gained popularity.

**Fig. 1:**
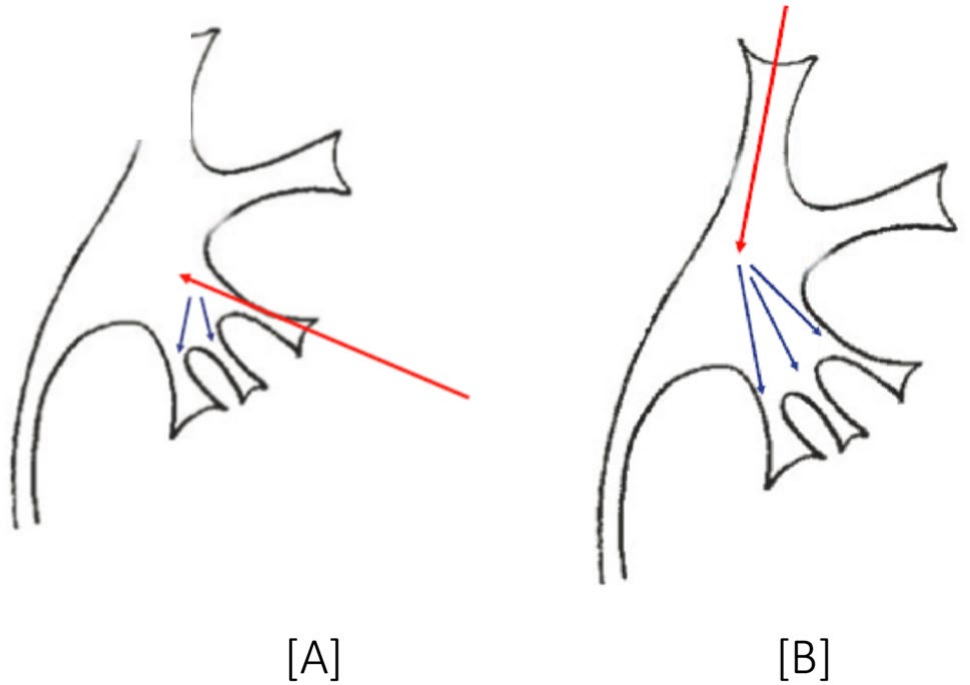
[9] Line diagrams showing. **a** Inferior calyceal Access: difficult angles to be negotiated to access multiple lower pole calyces. **b** Superior calyceal Access: Easy access to multiple inferior polar calyces

Superior calyceal access provides certain technical and anatomical advantages. The upper pole directed posteriorly makes it closer to the posterior flank wall in prone position, providing technically the shortest access tract. From an anatomical perspective the upper calyx is usually drained by a single calyceal infundibulum in the majority of the patients as shown by Sampaio et al. [8, 15] The superior calyx gives a straight access to all calyces and PUJ at minimal angulation, providing excellent visualization of the superior calyx, pelvis, ureter and the anterior and posterior inferior calyces (Fig. 1) [9]. The ease of operability has found to be more in the superior calyceal group, as the guidewire comfortably enters the pelvicalyceal system during the primary puncture. This is also confirmed in the present series (*p* = 0.034) and consistent with previous studies [9]. In the Inferior calyceal access for a pelvic stone or impacted stone in the lower calyx, slippage, bending and kinking of the guidewire is seen when passed through the percutaneous puncture needle and often making tract dilatation difficult. This leads to guidewire migration, bending, slippage and under dilatation of the tract. In the present series, 6.34% patients in inferior calyceal access group needed a repeat puncture due to guidewire dislodgement and displacement. This technical difficulty may be responsible for more bleeding and increased operative time which has not been studied by previous authors. Easy manipulation of the nephroscope into distal calyces and upper ureter through the superior calyceal access leads to minimal torque on the parenchyma and, therefore, minimal bleeding and facilitates removal of fragments migrating during fragmentation to other calyces and/or upper ureter [9]. Intrarenal navigation through the lower pole calices is associated with angulation and torque on the pelvicalyceal system often leading to unnecessary trauma and bleeding that may be avoided if access is established through the superior calyx.[7–12]. In this study, only subjective assessment of torque was made depending on the increased bleeding seen during the intrarenal navigation and the need to increase the irrigation inflow to maintain adequate vision. However, objective assessment of torque was not done and has also not reported in studies on PCNL.

In our study group I had larger sized access compared to group II. Since the superior calyx is a compound calyx that in the majority caused the surgeon to go for larger tract dilation. This will allow for an easy puncture, comfortable dilatation and achieve a fast stone clearance. A shorter surgery time in our study is probably related to the larger sized access used in 51 patients (80.9%) in both the groups with an Amplatz sheath size ranging between 28 and 32 Fr. The ease of calyceal access and dilatation made the stone clearance an easy exercise and thereby complete stone clearance rate was significantly higher in the superior calyceal access group (96.82%) when compared to the inferior calyceal group (85.71%). Stone free rates from isolated pelvic and lower calyceal calculi were significantly higher in superior calyceal group than inferior calyceal group. Overall, stone clearance rates are consistent with previous studies for superior calyceal access [2, 9, 13].

Although not statistically significant, the overall complication rate as classified by the modified Clavien grading system [14] was higher in the inferior calyceal group (17.4%) as compared to the superior calyceal group (11.1%). A similar trend was noted with higher rates of complications in the inferior calyceal group in several other studies [2, 9]. On the contrary in one study [13] complication rate was slightly higher in the superior calyceal group as compared to inferior calyceal group although not clinically significant.

In the previous study [9] residual stones that were demanding a second-look procedures were reported higher in the inferior calyceal group which was statistically significant (2% versus 18%). In the present study, the need for auxiliary procedures was slightly higher in the inferior calyceal access group although not statistically significant. This establishes a non-inferiority of superior calyceal access in achieving stone clearance rates.

## Limitations of our study

Since this is a prospective observational study there is an obvious lack of randomization. Moreover, the postoperative evaluation is only performed with KUB without NCCT scan. This is a well-known limitation in detecting RFs and CIRFs. However, the vast majority of similar studies in the literature have comparable follow up protocols. The reason for this is cost related, since this is a significant factor in the developing world.

## Conclusion

Superior calyceal puncture offers an easy access, dilatation and intrarenal navigation into most of the calyces. Superior calyceal access is better than for the inferior calyceal access. One can achieve a complete and faster stone clearance with fewer punctures, shorter procedural time, minimal risk of complications and less need for auxiliary procedures with minimal incidence of injury to the calyces. Surgeons may prefer to do upper calyx puncture because of easy access to the compound upper calyx, comfortable removal of lower calyceal stones with or without fragmentation with minimal infringement to the lower calyx. Stones in the pelvis can be managed by either the superior or inferior calyceal access based on personal preference but superior calyceal access is associated with better stone clearance.
